# Developmental Milestones in Young Transgender Women in Two American Cities: Results from a Racially and Ethnically Diverse Sample

**DOI:** 10.1089/trgh.2019.0008

**Published:** 2019-08-07

**Authors:** Arjee Restar, Harry Jin, Aaron Samuel Breslow, Anthony Surace, Nadav Antebi-Gruszka, Lisa Kuhns, Sari L. Reisner, Robert Garofalo, Matthew J. Mimiaga

**Affiliations:** ^1^ Department of Behavioral and Social Sciences, Brown University School of Public Health, Providence, Rhode Island.; ^2^ Department of Epidemiology, Brown University School of Public Health, Providence, Rhode Island.; ^3^ Center for Health Equity Research, Brown University, Providence, Rhode Island.; ^4^ PRIME Center for Health Equity, Albert Einstein College of Medicine, Bronx, New York.; ^5^ Health Equity Research Lab, Harvard Medical School, Cambridge, Massachusetts.; ^6^ Department of Psychology, Columbia University, New York, New York.; ^7^ Division of Adolescent Medicine, Ann and Robert H. Lurie Children's Hospital, Chicago, Illinois.; ^8^ Department of Pediatrics, Feinberg School of Medicine, Northwestern University, Chicago, Illinois.; ^9^ Division of General Pediatrics, Boston Children's Hospital/Harvard University Medical School, Boston, Massachusetts.; ^10^ Department of Epidemiology, Harvard T.H. Chan School of Public Health, Boston, Massachusetts.; ^11^ The Fenway Institute, Fenway Health, Boston, Massachusetts.

**Keywords:** gender development, milestones, transgender women, young

## Abstract

To understand developmental milestones among young transgender women (YTW), we mapped age estimates per milestone by race/ethnicity and cohort age using baseline data from Project Lifeskills (*n*=298). Compared with older and white participants, younger black, Latina, Asian, and other/mixed race transgender (trans) women reported earlier experiences of sexual debut, transfeminine identity disclosure to others, sexual debut as trans, transfeminine identity expression in public, and integration of hormone use. Findings call for increased research and utilization of gender-affirmative interventions among YTW, with incorporation of nuanced, intersecting roles of race/ethnicity and cohort age across milestones.

## Introduction

The processes by which young transgender women (YTW; ages 16–29 years) develop and express a transfeminine identity remain largely uncharacterized^1,2^ Such knowledge is critical, given that increasing numbers of YTW present for gender-affirmative care,^3^ and an overwhelming majority report disproportionate rates of mental health problems (e.g., suicidal behavior)^4–6^ and sexually transmitted infections (STIs; e.g., HIV) within this age group.^7–10^ It is important to understand how YTW, early in life, navigate complex personal and social milestones to provide affirmative interventions at appropriate developmental milieus addressing stigma, sexual health, and social support.

Within the transgender (trans) health literature, a short list of developmental models has mainly focused on transfeminine identity in adults using qualitative methods to describe stages of development.^11–15^ For example, one study^11^ adapted a gay and lesbian model of the “coming-out” processes for trans adults, detailing specific milestones such as initial awareness of identity, disclosure of identity to others, transition/body modification, and societal integration and acceptance. Another study using a transsexual identity formation model^12^ posited 14 stages that center on the process of external validation of gender through witnessing and mirroring others. In a qualitative study using a life course developmental framework,^13^ three stages of gender development were identified, including dissonance of body and mind, identity management, and transitioning for those who choose to undergo medical gender affirmation.

Across studies, developmental models face common limitations^16^; the majority, for example, (1) were adapted from gay/lesbian identity developmental paradigms, (2) conflate sexual orientation and transfeminine identity through frameworks of lesbian, gay, bisexual, and transgender identity development, (3) draw from relatively small samples, (4) tend not to have data specific to YTW, (5) and do not consider the impact of demographic differences (e.g., race/ethnicity, age cohort) in their analysis. This study aimed to address these limitations by mapping age estimates of developmental milestones for YTW by race/ethnicity and cohort age in a racially/ethnically diverse community sample.

## Methods

Participants were 298 YTW recruited in Chicago, Illinois, and Boston, Massachusetts, to participate in Project LifeSkills, a randomized controlled trial to reduce risk for HIV acquisition and transmission for YTW.^17^ The study's protocol has been described in detail elsewhere.^18^ Participants were recruited using multiple sampling strategies, including venue-based sampling, study flyers, and snowball recruitment. Eligibility requirements included the following: (1) being 16–29 years old; (2) assigned male sex at birth and current gender identity on the transfeminine spectrum (e.g., transgender woman, trans woman, woman, male-to-female); (3) self-reported engagement in condomless anal sex with an HIV status discordant/unknown partner in the past 4 months; and (4) being proficient in English. Participants completed a 2-h baseline study visit that included a computer-assisted survey to collect demographic characteristics (i.e., race/ethnicity, age, employment status, self-identified sexual orientation, and educational attainment) and developmental milestones. All participants completed written informed consent. The study was approved by the Institutional Review Boards at Lurie Children's Hospital/Fenway Health.

To assess YTW's developmental milestones (listed in Table 2), this study asked participants the age (continuous) when they first became self-aware of their transfeminine identity; disclosed their transfeminine identity to others; expressed their transfeminine identity privately and publicly; and had consensual receptive oral/vaginal/anal sex that involved a cisgender male sexual partner both before and after identifying on the transfeminine spectrum. Participants were also asked if they had ever taken feminizing hormones (yes/no) and the age they first began taking feminizing hormones (i.e., hormone integration).

We compared demographic characteristics and developmental milestones by race/ethnicity (i.e., black, Latina, Asian, white, and other) and cohort age categories (i.e., 16–20, 21–25, and 25–29 years). Analysis of variance and chi-square tests were conducted to identify differences in demographic characteristics and milestones between racial/ethnic and cohort age groups. All analyses were conducted in SAS 9.4.^19^

## Results

### Sample characteristics

Table 1 includes demographic characteristics and differences of these by race/ethnicity. At the point of data collection, approximately half of the sample reported their race as black (48.99%), between the ages of 21 and 25 years (45.30%), and mostly self-identified as heterosexual (39.26%); nearly three-quarters reported being unemployed (74.16%). Racial/ethnic differences were identified for age at baseline, study site, employment status, and educational attainment.

**Table 1.** Demographic Characteristics of the Study Sample of Young Transgender Women by Racial/Ethnic Composition (*n*=298)

	Total, *n* (%)	Black, *n* (%)	Latina, *n* (%)	Asian, *n* (%)	Other,^a^*n* (%)	White, *n* (%)	χ^2^*p*-value
Age in years at baseline	298 (100.0)	146 (48.99)	37 (12.42)	7 (2.35)	32 (10.74)	76 (25.50)	<0.0001^***^
16–20	87 (29.19)	52 (35.62)	13 (35.14)	3 (42.86)	7 (21.88)	12 (15.79)	
21–25	135 (45.30)	69 (47.26)	15 (40.54)	2 (28.57)	21 (65.63)	28 (36.84)	
26–29	76 (25.50)	25 (17.12)	9 (24.32)	2 (28.57)	4 (12.50)	36 (47.37)	
Study site							<0.0001^***^
Boston	145 (48.66)	36 (24.66)	24 (64.86)	7 (100.00)	14 (43.75)	64 (84.21)	
Chicago	153 (51.34)	110 (75.34)	13 (35.14)	0 (0.00)	18 (56.25)	12 (15.79)	
Self-identified Sexual orientation							<0.0001^***^
Gay/homosexual	79 (26.51)	50 (34.25)	11 (29.73)	1 (14.29)	11 (34.38)	6 (7.89)	
Lesbian	16 (5.37)	1 (0.68)	0 (0.00)	1 (14.29)	2 (6.25)	12 (15.79)	
Bisexual	58 (19.46)	23 (15.75)	8 (21.62)	1 (14.29)	4 (12.50)	22 (28.95)	
Heterosexual	117 (39.26)	63 (43.15)	17 (45.95)	2 (28.57)	12 (37.50)	23 (30.26)	
Other	28 (9.40)	9 (6.16)	1 (2.70)	2 (28.57)	3 (9.38)	13 (17.11)	
Employment							0.0079^**^
Yes	77 (25.84)	27 (18.49)	16 (43.24)	3 (42.86)	6 (18.75)	25 (32.89)	
No	221 (74.16)	119 (81.51)	21 (56.76)	4 (57.14)	26 (81.25)	51 (67.11)	
Education							<0.0001^***^
GED/high school, or less	175 (58.72)	99 (67.81)	28 (75.68)	1 (14.29)	20 (62.50)	27 (35.53)	
Trade school, college, or more	123 (41.28)	47 (32.19)	9 (24.32)	6 (85.71)	12 (37.50)	49 (64.47)	

^**^ *p*<0.01; ^***^p<0.001.

^a^ Other race/ethnicity include multiracial/mixed (e.g., black and Brazilian, black and white), Egyptian, Brazilian, and Haitian.

**χ**^2^, chi-square tests.

### Developmental milestones by race/ethnicity and cohort age

For this sample, the average age of initial awareness of transfeminine identity was between ages 9 and 10 years, with a standard deviation of 5 years. YTW reported initial private and public transfeminine gender expression within 2 and 7 years of self-identifying on the transfeminine spectrum, respectively. Subsequently, YTW on average reported their first sex at age 15 years, disclosed their transfeminine identity to others by age 15 years, and began hormone integration by age 20 years.

Table 2 includes the mean age at which participants reported completion of each developmental milestone by race/ethnicity and age cohort. Racial/ethnic and age cohort differences were identified for the mean age of each developmental milestone (all *p*<0.05). Overall, YTW of color (e.g., black, Latina, Asian, other/mixed race) in this sample and those who are in younger age cohorts reached each developmental milestone earlier in life compared with white women in the older age cohort.

**Table 2.** Gender Development Milestones in the Sample of Young Transgender Women by Race/Ethnicity and Cohort Age (Age at Baseline, *n*=298)

	Race/ethnicity						Cohort age (years)				
	Mean—all (*n*=298)	Black (*n*=146)	Latina (*n*=37)	Asian (*n*=7)	Other^a^ (*n*=32)	White (*n*=76)	F or χ^2^*p*-value	16–20 (*n*=87)	21–25 (*n*=135)	25–29 (*n*=76)	*F* or χ^2^*p*-value
Developmental milestones, in mean age (standard deviation)											
Initial self-awareness of transfeminine identity	9.91 (5.18)	10.84 (4.60)	8.82 (4.84)	10.14 (6.26)	9.43 (6.15)	9.04 (5.61)	0.1051	9.58 (4.92)	9.96 (5.14)	10.19 (5.6)	0.7776
Transfeminine identity disclosure to others	15.82 (5.75)	15.31 (5.44)	13.83 (4.78)	16.83 (2.93)	15.54 (5.45)	17.62 (6.52)	0.0121^*^	14.38 (4.34)	15.16 (5.43)	18.34 (6.69)	<0.0001^***^
Transfeminine expression in private	12.94 (5.36)	13.04 (5.11)	12.56 (4.48)	12.83 (6.43)	12.43 (5.67)	13.14 (6.02)	0.9670	11.93 (4.91)	13.19 (5.41)	13.57 (5.67)	0.1433
Transfeminine expression in public	17.41 (4.53)	16.24 (4.31)	15.88 (2.64)	18.57 (3.31)	17.31 (3.80)	19.96 (4.89)	<0.0001^***^	15.41 (3.53)	17.00 (3.97)	20.10 (5.01)	<0.0001^***^
First consensual oral/vaginal/anal sex with a cisgender male partner	14.92 (4.43)	13.30 (4.19)	14.69 (3.72)	17.00 (1.79)	15.65 (3.39)	17.79 (4.37)	<0.0001^***^	13.36 (4.28)	14.96 (3.82)	16.68 (4.96)	<0.0001^***^
First consensual oral/vaginal/anal sex with a cisgender male partner as a trans woman	16.68 (5.20)	14.70 (5.23)	15.80 (2.99)	18.20 (2.86)	17.13 (3.55)	20.79 (4.36)	<0.0001^***^	14.41 (4.88)	16.51 (4.84)	19.25 (5.02)	<0.0001^***^
Hormone utilizations											
Ever took hormones (yes)	213 (71.48)	101 (69.18)	29 (78.38)	5 (71.43)	21 (65.63)	57 (75.00)	0.6912	48 (55.17)	96 (71.11)	69 (90.79)	<0.0001^***^
Hormone integration (in age)	20.40 (4.04)	19.01 (3.99)	20.07 (3.39)	21.50 (2.89)	20.53 (3.32)	22.75 (3.76)	<0.0001^***^	17.78 (1.36)	19.82 (3.62)	23.16 (4.33)	<0.0001^***^

^*^ *p*<0.05; ^***^p<0.001.

^a^ Other race/ethnicity include multiracial/mixed (e.g., black and Brazilian, black and white), Egyptian, Brazilian, and Haitian.

F, analysis of variance tests; χ^2^, chi-square tests.

## Discussion

The findings of this study, to our knowledge, represent the first empirical quantitative account of YTW's developmental milestones. Specifically, we described age estimates when each milestone occurred, missing from previous studies.^11–15^ In addition, our findings indicate that while private/internal developmental milestones (e.g., self-awareness of transfeminine identity) do not significantly differ by race/ethnicity or cohort age, the social and external milestones (e.g., transfeminine identity disclosure to others,) do differ. Specifically, younger black, Latina, Asian, and other/mixed race participants experienced these social and external milestones earlier compared with older and white participants. This supports previous findings that other factors (e.g., racial discrimination, norms of concealment/disclosure) may precipitate YTW of color to meet such social/external milestones earlier relative to white YTW.^20^ These results suggest there is a need for integrated gender-, youth-, and race/ethnicity-affirmative early-in-life interventions, especially when YTW of color transition from more internal recognition of transfeminine identity to more social expression processes of disclosure and sexual emergence.^21^ That is, although reaching these external milestones early may be empowering for YTW of color, such rapid development may put such YTW at elevated risk given the dual impact of gender and racial minority marginalization.^4^ As such, results call for early-in-life interventions supporting YTW through these social and external milestones of development. Interventions may incorporate resilience, social support, and sexual risk reduction to better equip YTW with effective means to navigate the immense challenges they face in these periods of transition.^22^

Moreover, we also found that the age of first consensual oral/anal/vaginal sex among this sample is lower than the U.S. average in cisgender (non-transfeminine) women (15 vs. 17 years old),^23,24^ an indication for the need of early-in-life sexual health and risk prevention interventions. In particular, YTW of color, who may be particularly vulnerable to HIV/STI, reported sexual debut at roughly 13 years of age, earlier than both white YTW (18 years old) and the U.S. average for cisgender women (17 years old). As previous research has shown, age of first sexual experience is predictive of HIV/STI risk,^25^ and that TW of color are disproportionately impacted by HIV/STIs.^9^ These results underscore that early-in-life sexual health interventions that pay closer attention to the age of first sex are needed, which may be useful in reducing the risk of HIV/STI among YTW. Furthermore, it is important to situate age of first consensual sexual encounter (e.g., oral/anal/vaginal sex) within the context of internal and external gender identity milestones for YTW.

There are some limitations to the current analyses. First, the eligibility criteria are from a randomized control study that aimed to lower HIV risk behaviors among YTW, which may limit the finding's generalizability as the study sample on enrollment was “high risk.” That is, utilizing participants recruited for a clinical trial may not represent the overall population of YTW. Second, due to the limitations of the Project Lifeskills study design, only a small developmental window and set of developmental milestones could be examined, future research should expand on this work to elucidate the detailed milestones of transfeminine identity development across the lifetime, as well as different types of sexual partners of YTW besides cisgender men. Furthermore, data are cross-sectional and therefore do not indicate causal relationships between race/ethnicity, age, and developmental milestones. Third, *post hoc* tests were not conducted, which limits confirmation as to how each race/ethnic and cohort age group specifically differs from one another. Fourth, the data were collected from English-speaking YTW across two cities within the United States; as such, our findings have limited generalizability across regions/cultures. Finally, data collection relied on participants to recall age of specific developmental milestone completion, which may be inaccurate; future studies should utilize other valid recall methods.

The current study, despite its limitations, does provide insight into YTW's development and does raise questions for future research. Namely, future research should expand on this work by identifying contributing factors for the earlier experiences of first consensual sexual encounter, hormone use, and disclosure among YTW. In addition, research is needed on the strengths and resilience of YTW so as to develop interventions that foster positive youth development. Furthermore, future research is needed on the impact of intersectionality in explaining the disparities found in this study. Finally, due to Project Lifeskills' focus on YTW, it is unclear how these results apply to other gender minority populations (e.g., nonbinary individuals). Future research is needed to clarify how various gender minorities develop across the life span.

Along with research implications, these results suggest the need for health programs/interventions that incorporate content relevant to YTW's development. Such multilevel programs may include the following: (1) policy recommendations that promote greater acceptance and eliminate discrimination on YTW while in school (e.g., anti-bullying policy, gender and sexuality alliances); (2) easier and safer access to hormones, especially for YTW of color; (3) trans-affirmative counseling efforts that focus on identity formation, self-acceptance, disclosure, and resilience; (4) trans-affirmative (e.g., emphasize that gender identity is diverse and fluid, and that no gender expression is pathological^26^) sex education and sexual health programming in school settings; (5) greater family support for YTW, particularly in critical milestones of their development; and (6) in-depth understanding of the contribution of race/ethnicity and age in YTW's development for health care providers.

## Abbreviations Used

STIs: sexually transmitted infections
trans: transgender
YTW: young transgender women
